# Independent Predictors of Symptomatic Hypocalcemia Following Thyroidectomy: Distinguishing Biochemical and Symptomatic Hypocalcemia

**DOI:** 10.3390/jcm15197522

**Published:** 2026-09-27

**Authors:** Furkan Türkoğlu, Muhammed Gürlük, Ömer Akay, Candeniz Ertürk, Elif Aydın Elçi, Cebrail Oğuz, Elif Nur Gencer, Ufuk Oğuz İdiz

**Affiliations:** 1 Department of General Surgery, Aktif International Hospital, 41275 Kocaeli, Türkiye; 2 Department of General Surgery, Bingöl Genç State Hospital, 12500 Bingöl, Türkiye; mgurluk12@gmail.com; 3 Department of General Surgery, Kocaeli City Hospital, 41275 Kocaeli, Türkiye; omer_akay_38@hotmail.com; 4 Department of General Surgery, Kahramanmaraş State Hospital, 46040 Kahramanmaraş, Türkiye; candenizerturk@gmail.com; 5 Department of General Surgery, Health Sciences University, Istanbul Training and Research Hospital, 34098 İstanbul, Türkiye; elifaydn4450@gmail.com (E.A.E.); ufukidiz@gmail.com (U.O.İ.); 6 Department of General Surgery, Bitlis State Hospital, 13100 Bitlis, Türkiye; dr.cebrailoguz@gmail.com; 7 Department of General Surgery, Tuzla State Hospital, 34947 İstanbul, Türkiye; elifnurkocmar@yahoo.com

**Keywords:** thyroidectomy, symptomatic hypocalcemia, asymptomatic biochemical hypocalcemia, central neck dissection, parathyroid hormone, serum calcium, risk stratification, fine-needle aspiration biopsy

## Abstract

**Background/Objectives**: Postoperative hypocalcemia is the most common complication following thyroidectomy; however, previous studies have largely relied on biochemical definitions without distinguishing symptomatic hypocalcemia from asymptomatic biochemical abnormalities. This study aimed to identify the independent predictors of symptomatic postoperative hypocalcemia. **Methods**: This retrospective single-center study included 245 adult patients who underwent thyroidectomy between January 2017 and December 2022. Patients were classified as normocalcemic (*n* = 88), asymptomatic biochemical hypocalcemic (*n* = 87), or symptomatic hypocalcemic (*n* = 70) according to calcium status within the first 72 postoperative hours. Demographic, surgical, biochemical, ultrasonographic, cytological, and histopathological variables were analyzed. Independent predictors were identified using multivariable logistic regression. **Results**: Symptomatic hypocalcemia occurred more frequently after bilateral total thyroidectomy with central neck dissection (*p* = 0.001). Patients with symptomatic hypocalcemia had lower preoperative serum calcium and lower postoperative albumin and parathyroid hormone levels than the other groups (all *p* < 0.05). Although high-risk FNAB cytology and malignant histopathology were associated with hypocalcemia in univariate analyses, neither remained independently predictive after adjustment. Bilateral total thyroidectomy with central neck dissection (adjusted OR 6.63, 95% CI 1.68–26.11; *p* = 0.007) and lower preoperative serum calcium (adjusted OR 0.46, 95% CI 0.22–0.94; *p* = 0.036) were independent predictors of symptomatic hypocalcemia after thyroidectomy. **Conclusions**: Symptomatic postoperative hypocalcemia should be considered a distinct clinical outcome rather than merely a biochemical abnormality. Bilateral total thyroidectomy with central neck dissection and lower preoperative serum calcium independently predicted symptomatic hypocalcemia, whereas ultrasonographic findings, FNAB cytology, histopathological diagnosis, and incidental parathyroidectomy did not. Although the absence of perioperative vitamin D and magnesium measurements is an important limitation, the identified predictors may provide clinically useful information for perioperative risk stratification.

## 1. Introduction

Thyroidectomy is among the most commonly performed endocrine surgical procedures worldwide and remains the cornerstone of treatment for both malignant and selected benign thyroid diseases [1,2,3]. Over the past three decades, the global incidence of thyroid cancer has increased substantially, largely owing to advances in high-resolution ultrasonography, widespread implementation of fine-needle aspiration biopsy (FNAB), and improved detection of early-stage thyroid malignancies [3]. Consequently, an increasing number of patients undergo thyroidectomy not only for thyroid cancer but also for multinodular goiter, compressive goiter, toxic thyroid disease, and indeterminate thyroid nodules requiring definitive diagnosis [1,2]. As the volume of thyroid surgery continues to rise, minimizing procedure-related complications has become an increasingly important objective in endocrine surgical practice.

Despite continuous refinements in surgical techniques and perioperative care, postoperative hypocalcemia remains the most common complication following thyroidectomy [4]. Reported rates of transient hypocalcemia range from approximately 20% to 40%, whereas permanent hypoparathyroidism occurs in up to 3% of patients [5,6,7]. Although transient hypocalcemia is usually self-limiting, it may prolong hospitalization, increase healthcare costs, necessitate calcium and vitamin D supplementation, and adversely affect patients’ quality of life. Moreover, postoperative hypocalcemia remains one of the principal barriers to early discharge following thyroidectomy and represents a major determinant of postoperative resource utilization. Permanent hypoparathyroidism, although less common, requires lifelong medical therapy and biochemical surveillance, making preservation of parathyroid gland function one of the primary goals of thyroid surgery.

The development of postoperative hypocalcemia is multifactorial. Temporary or permanent impairment of parathyroid hormone secretion resulting from inadvertent parathyroidectomy, disruption of the delicate vascular supply to the parathyroid glands, excessive surgical manipulation, thermal injury, or postoperative edema has long been recognized as the principal mechanism underlying postoperative calcium imbalance. However, postoperative calcium homeostasis is also influenced by patient-related factors, preoperative biochemical status, thyroid pathology, and operative complexity. Despite extensive investigation, the relative contribution of these factors remains controversial, and reliable identification of patients at increased risk of postoperative hypocalcemia continues to represent a major clinical challenge [4,7,8,9].

Importantly, considerable heterogeneity exists in the definition of postoperative hypocalcemia across the literature [5,6,7,8,9,10]. Many previous studies have relied primarily on biochemical thresholds based on serum calcium concentrations, whereas others have combined laboratory findings with clinical manifestations or focused on postoperative parathyroid hormone kinetics alone. Consequently, asymptomatic biochemical hypocalcemia and symptomatic hypocalcemia are frequently analyzed together despite their different clinical implications. While isolated biochemical hypocalcemia often requires only observation, symptomatic hypocalcemia directly influences postoperative management by prompting calcium replacement therapy, extending hospitalization, and delaying discharge. Distinguishing symptomatic hypocalcemia from asymptomatic biochemical abnormalities may therefore provide more clinically meaningful risk stratification and improve postoperative decision-making.

Accurate prediction of clinically relevant postoperative hypocalcemia has important implications for modern endocrine surgery [10]. Early identification of high-risk patients may facilitate individualized postoperative monitoring, support selective rather than routine calcium supplementation, reduce unnecessary laboratory testing, and enable safe early discharge. Nevertheless, relatively few studies have simultaneously evaluated the broad spectrum of demographic, biochemical, ultrasonographic, cytological, histopathological, and surgical factors while distinguishing symptomatic hypocalcemia from asymptomatic biochemical hypocalcemia within the same patient cohort.

Therefore, the present study aimed to identify clinically relevant predictors of early postoperative hypocalcemia following thyroidectomy, with particular emphasis on symptomatic hypocalcemia rather than biochemical abnormalities alone. By differentiating symptomatic from asymptomatic postoperative calcium disturbances, we sought to provide a clinically meaningful framework for perioperative risk stratification and individualized postoperative calcium management.

## 2. Materials and Methods

### 2.1. Study Design and Ethical Approval

This retrospective, single-center observational study was conducted at a tertiary referral center. The study protocol was approved by the Local Clinical Research Ethics Committee (Decision No. 2011-KAEK-50-183; approval date: 21 July 2023) and was performed in accordance with the ethical principles of the Declaration of Helsinki. The study was designed and reported in accordance with the Strengthening the Reporting of Observational Studies in Epidemiology (STROBE) statement.

Written informed consent for the surgical procedure had been obtained from all patients as part of routine clinical practice. Owing to the retrospective nature of the study and the use of fully anonymized data, the requirement for additional informed consent for study participation was waived by the ethics committee.

ChatGPT (GPT-5.5, OpenAI, San Francisco, CA, USA) was used exclusively for minor language editing and stylistic refinement to improve the clarity, grammar, and readability of the manuscript. It was not involved in the conception or design of the study, data acquisition, statistical analyses, interpretation of the findings, or formulation of the scientific conclusions. All scientific content, interpretation of the findings, and final decisions were made exclusively by the authors, who assume full responsibility for the accuracy and integrity of the manuscript.

### 2.2. Patient Selection

Hospital information systems, institutional clinical archives, and the endocrine surgery database were retrospectively reviewed to identify consecutive adult patients who underwent thyroidectomy between January 2017 and December 2022. Patients aged 18 years or older who underwent thyroidectomy and had complete clinical and biochemical follow-up during the early postoperative period were considered eligible for inclusion. Patients with advanced chronic kidney disease or other chronic diseases associated with clinically relevant electrolyte disturbances, as well as patients with symptomatic electrolyte abnormalities or electrolyte disturbances requiring treatment before surgery, were excluded. Patients who underwent intraoperative parathyroid autotransplantation, had incomplete postoperative biochemical or clinical follow-up, or had missing essential medical records were also excluded.

### 2.3. Definition of Study Groups and Outcome Measures

The primary outcome of this study was symptomatic postoperative hypocalcemia. Because isolated biochemical hypocalcemia may not necessarily reflect clinically relevant parathyroid dysfunction, serum calcium concentrations alone were not considered sufficient for outcome classification. Instead, patients were categorized according to both postoperative biochemical findings and clinical manifestations into three groups: “normocalcemia”, defined as normal postoperative serum calcium levels without symptoms; “asymptomatic biochemical hypocalcemia”, defined as biochemical hypocalcemia in the absence of clinical manifestations; and “symptomatic postoperative hypocalcemia”, defined as biochemical hypocalcemia accompanied by at least one clinical manifestation occurring within the first 72 postoperative hours.

Biochemical hypocalcemia was defined as a total serum calcium concentration below the lower limit of the institutional laboratory reference range (<8.5 mg/dL). Total serum calcium measurements reported by the institutional clinical laboratory were used throughout the study.

Clinical manifestations of hypocalcemia included perioral numbness, acral paresthesia, muscle cramps, positive Chvostek sign, positive Trousseau sign, or other symptoms considered by the treating endocrine surgery team to be attributable to hypocalcemia. At the study institution, all patients remained hospitalized for at least 72 h after thyroidectomy and underwent routine clinical assessment by the endocrine surgery team twice daily, during morning and evening ward rounds. A systematic physical examination for clinical signs of hypocalcemia was performed at each assessment, and any detected symptoms or physical findings were documented in the patient’s daily clinical observation notes. This standardized assessment protocol, together with postoperative biochemical measurements, was used to classify patients according to their clinical and biochemical status during the first 72 postoperative hours.

### 2.4. Clinical Data Collection and Laboratory Assessment

Clinical and laboratory data were retrospectively retrieved from the hospital information system, institutional clinical archives, and the endocrine surgery database. Demographic and surgical information, including sex, surgical indication, type of thyroidectomy, and drain placement, was extracted from the medical records. Surgical procedures were categorized as unilateral thyroidectomy, bilateral total thyroidectomy, completion thyroidectomy, and bilateral total thyroidectomy with central neck dissection. Surgical indications were categorized as multinodular goiter, hyperthyroidism, obstructive or substernal goiter, papillary thyroid carcinoma, indeterminate thyroid nodules (Bethesda III; atypia of undetermined significance/follicular lesion of undetermined significance [AUS/FLUS]), follicular neoplasm or suspicion for follicular neoplasm, and other uncommon thyroid diseases requiring surgical treatment, including anaplastic thyroid carcinoma. Parathyroid glands were identified and preserved using standard surgical anatomical techniques. No specific intraoperative parathyroid detection or viability assessment technology, such as near-infrared autofluorescence or indocyanine green fluorescence, was routinely used during the study period. LigaSure vessel-sealing technology (Medtronic, Minneapolis, MN, USA) was routinely used for haemostasias during thyroidectomy.

All preoperative biochemical measurements were obtained within the final 24 h before surgery as part of the routine preoperative evaluation. The recorded laboratory parameters included total serum calcium, albumin, parathyroid hormone (PTH), thyroid-stimulating hormone (TSH), free triiodothyronine (T3), free thyroxine (T4), anti-thyroid peroxidase antibody (anti-TPO), and serum creatinine. Routine postoperative biochemical assessment was performed within the first 24–48 h after thyroidectomy and included total serum calcium, albumin, and PTH measurements. No routine calcium, magnesium, or vitamin D supplementation was administered preoperatively to any patient included in the study. Similarly, no routine prophylactic calcium or vitamin D supplementation was administered postoperatively.

### 2.5. Ultrasonographic, Cytological, and Histopathological Evaluation

Preoperative ultrasonographic findings were evaluated as part of the clinical and radiological assessment. Ultrasonographic variables recorded for analysis included the largest nodule diameter, nodule composition (solid, mixed, or cystic), echogenicity, margin characteristics, presence and pattern of calcifications, vascularity, and the Thyroid Imaging Reporting and Data System (TI-RADS) category.

Fine-needle aspiration biopsy (FNAB) was performed when clinically indicated and was available for 198 patients. Cytological findings were classified according to the Bethesda System for Reporting Thyroid Cytopathology as Bethesda I (nondiagnostic), Bethesda II (benign), Bethesda III (atypia of undetermined significance/follicular lesion of undetermined significance [AUS/FLUS]), Bethesda IV (follicular neoplasm or suspicion for a follicular neoplasm), Bethesda V (suspicious for malignancy), and Bethesda VI (malignant). For statistical analyses, FNAB results were further dichotomized into low-risk cytology (Bethesda categories I–II) and high-risk cytology (Bethesda categories III–VI), representing indeterminate or malignant cytological findings that generally warrant surgical management.

Final histopathological findings were evaluated and classified according to the definitive pathological diagnosis. Based on the definitive pathological diagnosis, patients were initially classified into six histopathological categories: (1) benign nodular disease (adenomatous hyperplasia and diffuse hyperplasia), (2) thyroiditis (Hashimoto thyroiditis, De Quervain thyroiditis, and granulomatous thyroiditis), (3) benign neoplasm (follicular adenoma and Hurtle cell adenoma), (4) papillary thyroid carcinoma, (5) medullary thyroid carcinoma, and (6) other malignant tumors, including follicular carcinoma, poorly differentiated carcinoma, and anaplastic thyroid carcinoma. For additional analyses, these categories were further dichotomized into benign (categories 1–3) and malignant (categories 4–6) pathological diagnoses.

Histopathological reports of all patients were reviewed to identify incidentally excised parathyroid glands, and both their presence and number were recorded as potential predictors of postoperative hypocalcemia.

### 2.6. Statistical Analysis

Statistical analyses were performed using IBM SPSS Statistics for Windows, Version 27.0 (IBM Corp., Armonk, NY, USA). Data distribution was assessed using both visual inspection (histograms and Q-Q plots) and analytical methods (Kolmogorov–Smirnov and Shapiro–Wilk tests). As all continuous variables demonstrated non-normal distributions, they are summarized as median (interquartile range [IQR]; minimum–maximum), whereas categorical variables are presented as frequencies and percentages.

Comparisons among the three study groups were performed using the Kruskal–Wallis test for continuous variables. When overall group differences were statistically significant, post hoc pairwise comparisons were conducted using the Mann–Whitney U test with Bonferroni correction for multiple testing. Categorical variables were compared using Pearson’s chi-square test when the assumptions for the test were satisfied. Fisher’s exact test was applied when expected cell counts were <5 in more than 20% of the contingency table or when sample sizes were insufficient to meet the assumptions of the chi-square test.

To identify independent predictors of symptomatic postoperative hypocalcemia, binary logistic regression analysis was performed using symptomatic hypocalcemia (yes/no) as the dependent variable. For this analysis, normocalcemia and asymptomatic biochemical hypocalcemia were combined as the reference category, whereas symptomatic hypocalcemia constituted the outcome of interest. This approach was chosen to identify predictors of clinically relevant symptomatic hypocalcemia rather than isolated biochemical calcium abnormalities. Variables demonstrating statistical significance in the univariate analyses or considered clinically relevant based on previous literature were simultaneously entered into the multivariable model using the enter method. Results are reported as adjusted odds ratios (ORs) with 95% confidence intervals (CIs). Model performance was evaluated using the Omnibus Test of Model Coefficients, Nagelkerke’s R^2^, and the classification accuracy of the final model. All statistical tests were two-sided, and a *p* value < 0.05 was considered statistically significant.

## 3. Results

A total of 245 patients who underwent thyroidectomy during the study period were included in the study. According to postoperative calcium status, 88 patients (35.9%) remained normocalcemic, 87 (35.5%) developed asymptomatic biochemical hypocalcemia, and 70 (28.6%) developed symptomatic hypocalcemia during the first 72 postoperative hours.

The distribution of surgical procedures differed significantly according to postoperative calcium status (*p* = 0.001). Bilateral total thyroidectomy with central neck dissection was most frequently observed among patients with symptomatic hypocalcemia, whereas unilateral thyroidectomy was most frequently observed in the normocalcemic group. Surgical indication and drain placement were not significantly associated with postoperative calcium status (Table 1).

Comparison of perioperative biochemical parameters demonstrated significant differences in preoperative serum calcium, early postoperative albumin, and early postoperative PTH levels among the study groups. Patients who developed symptomatic hypocalcemia had significantly lower preoperative calcium concentrations than normocalcemic patients. Likewise, postoperative albumin and PTH levels progressively decreased with increasing severity of postoperative calcium disturbance. In contrast, preoperative thyroid function tests, albumin, creatinine, anti-thyroid peroxidase antibody levels, and preoperative PTH concentrations did not differ significantly among the groups (Table 2).

Pairwise comparisons demonstrated that patients with normocalcemia had significantly higher preoperative serum calcium concentrations than both the hypocalcemic groups (*p* = 0.021 and *p* = 0.011, respectively). Similarly, postoperative albumin levels were significantly higher in the normocalcemia group than in the hypocalcemic groups (*p* = 0.013 and *p* = 0.001, respectively). In contrast, postoperative PTH levels were significantly lower in the symptomatic hypocalcemia group than in both the normocalcemia and asymptomatic biochemical hypocalcemia groups (*p* < 0.001 for both), whereas no difference was observed between the latter two groups (*p* = 0.178). Post hoc pairwise comparisons are presented in Table 3.

Preoperative thyroid ultrasonography was available for 227 of the 245 patients included in the study. Ultrasonographic characteristics, including TI-RADS classification, nodule size, echogenicity, calcification pattern, margin characteristics, and vascularity, were comparable among the study groups (*p* > 0.05 for all). None of the evaluated ultrasonographic variables demonstrated an association with the development of symptomatic hypocalcemia (Table 4).

Fine-needle aspiration biopsy cytological classification was available for 198 patients and differed among the study groups when analyzed according to the individual Bethesda categories (*p* = 0.047). However, after dichotomization into low-risk (Bethesda I–II) and high-risk (Bethesda III–VI) categories, no significant association was observed with symptomatic postoperative hypocalcemia (Table 5).

Final histopathological diagnoses differed significantly according to postoperative calcium status (*p* = 0.020) (Table 6). Patients who developed symptomatic hypocalcemia had a higher proportion of malignant thyroid pathology, particularly papillary thyroid carcinoma, whereas benign nodular disease was more frequently observed in the normocalcemic group. When final histopathological diagnoses were further dichotomized, malignant final histopathology was more frequent among patients with symptomatic hypocalcemia than among those with normocalcemia or asymptomatic biochemical hypocalcemia (*p* = 0.012). In contrast, the number of incidentally excised parathyroid glands identified on final histopathological examination did not differ significantly among the groups (*p* = 0.131).

Variables demonstrating statistical significance in the univariate analyses or considered clinically relevant were entered into a multivariable logistic regression model to identify independent predictors of symptomatic postoperative hypocalcemia. Because postoperative PTH and albumin levels are measured after surgery and represent early postoperative biochemical markers that lie within the causal pathway of postoperative calcium homeostasis, they were intentionally excluded from the multivariable model to avoid overadjustment. Furthermore, because the final histopathological diagnosis becomes available only after surgery and is highly correlated with the preoperative FNAB cytological classification, only the preoperative FNAB risk category was included in the multivariable model to preserve its clinical applicability as a predictive model and to avoid redundancy between closely related variables. The overall model was statistically significant (Omnibus χ^2^ = 15.668, *p* = 0.008), explaining 12.0% of the variance in the primary outcome (Nagelkerke R^2^ = 0.120). After adjustment, bilateral total thyroidectomy with central neck dissection remained independently associated with an increased risk of symptomatic hypocalcemia compared with unilateral thyroidectomy (adjusted OR 6.63, *p* = 0.007). In addition, higher preoperative serum calcium levels independently reduced the likelihood of developing symptomatic hypocalcemia (adjusted OR 0.46 per 1 mg/dL increase, *p* = 0.036). The preoperative FNAB cytological risk category was not independently associated with the primary outcome (Table 7).

## 4. Discussion

Postoperative hypocalcemia is a common complication following thyroidectomy and may substantially influence postoperative management and healthcare utilization. Although numerous studies have investigated the risk factors for postoperative hypocalcemia [5,6,7,8,9,10], the existing literature is characterized by substantial heterogeneity in the definition of this outcome, with studies variably relying on biochemical thresholds, clinical symptoms, or a combination of both. Consequently, direct comparison between studies remains challenging, and the reported predictors are not always readily applicable to clinical practice. Importantly, biochemical hypocalcemia does not invariably result in clinical manifestations, as total serum calcium reflects both ionized and protein-bound calcium and may decrease without a corresponding clinically relevant reduction in ionized calcium. Therefore, isolated reductions in serum calcium may not require therapeutic intervention in the absence of symptoms [11,12]. Accordingly, considering symptomatic hypocalcemia as a distinct clinical outcome may provide a more clinically meaningful approach to risk stratification and individualized postoperative management. The present study therefore specifically focused on symptomatic hypocalcemia rather than isolated biochemical abnormalities. This clinically oriented classification represents one of the principal strengths of our study. The main findings were that bilateral total thyroidectomy with central neck dissection and lower preoperative serum calcium concentrations independently predicted symptomatic hypocalcemia, whereas preoperative ultrasonographic characteristics, FNAB cytology, final histopathological diagnosis, and incidental parathyroidectomy were not independently associated with the primary outcome.

A pivotal element of this study is the differentiation between asymptomatic biochemical and symptomatic hypocalcemia. Mild reductions in serum calcium concentrations are frequently observed after thyroidectomy and may resolve spontaneously without requiring treatment [13]. Consequently, classifying all cases of biochemical hypocalcemia as postoperative complications may overestimate their true clinical significance. White et al. [14] similarly demonstrated that biochemical hypocalcemia was not predictive of symptomatic hypocalcemia in the immediate postoperative period. The present study was specifically designed to distinguish patients with asymptomatic biochemical hypocalcemia from those who developed symptomatic hypocalcemia within the first 72 postoperative hours. By adopting this clinically oriented classification, our analyses focused on identifying the predictors and independent risk factors for symptomatic hypocalcemia, rather than for biochemical hypocalcemia alone. This approach provides a more clinically relevant framework for postoperative risk stratification and may better inform individualized patient management following thyroidectomy.

The extent of thyroid surgery emerged as one of the strongest determinants of symptomatic hypocalcemia in the present study. Although demographic and perioperative variables, including sex, surgical indication, and drain placement, were not associated with either asymptomatic or symptomatic hypocalcemia, the type of thyroidectomy differed significantly according to postoperative calcium status. In particular, patients undergoing more extensive surgical procedures experienced a substantially higher incidence of symptomatic hypocalcemia. While bilateral total thyroidectomy alone showed only a nonsignificant trend toward an increased risk, bilateral total thyroidectomy combined with central neck dissection remained an independent predictor in the multivariable model, increasing the odds of symptomatic hypocalcemia more than six-fold. This observation is biologically plausible because central neck dissection requires more extensive dissection in close proximity to the parathyroid glands and their delicate vascular supply, thereby increasing the likelihood of transient parathyroid dysfunction even when the glands are anatomically preserved. Previous systematic reviews and meta-analyses have consistently identified central neck dissection as one of the strongest surgical risk factors for postoperative hypocalcemia [15,16,17]. Our findings extend previous evidence by demonstrating that bilateral total thyroidectomy with central neck dissection is an independent predictor of symptomatic postoperative hypocalcemia, reinforcing the clinical value of distinguishing symptomatic hypocalcemia from isolated biochemical abnormalities.

Another important finding of the present study was the association between perioperative biochemical parameters and postoperative calcium status. In the univariate analyses, preoperative serum calcium, postoperative albumin, and postoperative PTH levels differed significantly among the study groups. Pairwise comparisons demonstrated that patients with normocalcemia had significantly higher preoperative calcium and postoperative albumin levels than both the asymptomatic and symptomatic hypocalcemia groups, whereas no significant differences were observed between the latter two groups. In contrast, postoperative PTH levels differed exclusively in patients who developed symptomatic hypocalcemia, with significantly lower concentrations than those observed in both the normocalcemia and asymptomatic hypocalcemia groups. Interestingly, postoperative PTH did not distinguish patients with asymptomatic biochemical hypocalcemia from those with normocalcemia, but clearly differentiated patients who developed symptomatic hypocalcemia. This finding further supports the concept that postoperative PTH primarily reflects clinically relevant parathyroid dysfunction rather than isolated biochemical calcium abnormalities. By comparison, postoperative albumin appeared to be associated with biochemical calcium changes regardless of symptom status, suggesting a less specific relationship with clinically relevant hypocalcemia. On the other hand, postoperative PTH and albumin were intentionally excluded from the multivariable model because both represent longitudinal biochemical markers within the causal pathway of postoperative calcium homeostasis rather than independent baseline risk factors [14,18,19]. Their inclusion could therefore result in overadjustment and obscure the identification of true predictors of symptomatic hypocalcemia. Moreover, because these parameters become available only after surgery, they have limited value for preoperative risk stratification. After adjustment for potential confounders, higher preoperative serum calcium concentration remained an independent protective factor against symptomatic hypocalcemia. Each 1 mg/dL increase in preoperative serum calcium concentration was independently associated with a 54% reduction in the odds of symptomatic postoperative hypocalcemia (adjusted OR, 0.46; 95% CI, 0.22–0.94; *p* = 0.036). Although serum calcium is tightly regulated under physiological conditions, lower preoperative calcium concentrations may reflect reduced physiological reserve, subclinical vitamin D deficiency, impaired calcium homeostasis, or limited functional reserve of the parathyroid glands before surgery. Previous studies have similarly suggested that patients presenting with lower baseline serum calcium concentrations or vitamin D deficiency are more susceptible to postoperative calcium disturbances [20,21,22,23]. However, several recent studies have challenged the predictive value of vitamin D status. In 2021, Soares et al. [24] reported that preoperative vitamin D deficiency was not an independent predictor of postoperative hypocalcemia, and similar findings were subsequently reported by Jan et al. [25] in 2024. These conflicting results underscore the complexity of calcium homeostasis after thyroidectomy. Rather than being determined by a single biochemical parameter, postoperative calcium balance appears to reflect the dynamic interplay between vitamin D status, parathyroid hormone secretion, calcium reserve, renal handling, and other metabolic and surgical factors. Against this background, the independent predictive value of preoperative serum calcium observed in our study is particularly noteworthy. Because preoperative serum calcium is inexpensive, universally available, and routinely measured before thyroidectomy, it represents a simple and practical biomarker for preoperative risk stratification. Nevertheless, because perioperative vitamin D concentrations were unavailable, the interaction between vitamin D status, parathyroid reserve, and postoperative calcium homeostasis could not be explored further. Future prospective studies incorporating comprehensive vitamin D assessment may help clarify these complex metabolic interactions.

One of the distinctive aspects of the present study was the comprehensive evaluation of preoperative imaging and cytological findings as potential predictors of symptomatic hypocalcemia. While previous studies have primarily focused on biochemical parameters, surgical factors, and postoperative PTH kinetics, relatively few have investigated whether preoperative USG characteristics or FNAB findings contribute to postoperative calcium risk stratification [26,27]. In the present study, none of the evaluated ultrasonographic variables were associated with postoperative calcium status. Likewise, although high-risk FNAB cytology was more frequently observed among patients who developed symptomatic hypocalcemia in the univariate analysis, it did not remain independently associated with symptomatic hypocalcemia after multivariable adjustment. The available literature regarding the influence of preoperative USG and cytological findings on postoperative hypocalcemia remains scarce and inconsistent. Halwani et al. [26], in a retrospective cohort of 260 patients, reported that thyroid nodules larger than 4 cm were associated with an increased risk of postoperative hypocalcemia. In contrast, Yazıcıoğlu et al. [27] found no association between thyroid nodule size and postoperative PTH concentrations. Our findings are more consistent with the latter observation, suggesting that preoperative imaging characteristics alone have limited value for predicting postoperative calcium disturbances. The absence of an independent association between FNAB cytology and symptomatic hypocalcemia is also clinically relevant. Although malignant or indeterminate cytology may increase the likelihood of more extensive surgical procedures, the cytological diagnosis itself does not appear to directly influence postoperative calcium homeostasis. Rather, its apparent effect is largely mediated through surgical extent and disease severity. Accordingly, FNAB cytology did not provide incremental predictive value beyond established clinical and surgical variables in the multivariable model. These findings indicate that routine preoperative ultrasonographic and cytological characteristics should not be considered independent risk factors for symptomatic postoperative hypocalcemia, although they remain essential for surgical planning and oncological decision-making.

Final histopathological examination demonstrated a higher proportion of malignant disease among patients who developed symptomatic hypocalcemia in the univariate analysis. This finding is consistent with previous reports suggesting that thyroid malignancy is associated with an increased risk of postoperative hypocalcemia. Similarly, Yazıcıoğlu et al. [27] reported a higher incidence of postoperative hypoparathyroidism among patients undergoing surgery for papillary thyroid carcinoma. The observed association may partly reflect the greater frequency of more extensive surgical procedures among patients with malignant disease, as extensive thyroid surgery and central neck dissection may increase the risk of transient parathyroid dysfunction. However, the specific indications for central neck dissection were not systematically available in the retrospective dataset and therefore could not be reliably evaluated. Accordingly, the relationship between malignancy and symptomatic hypocalcemia should be interpreted cautiously and should not be considered independent of the extent of surgery. Definitive histopathological diagnosis was intentionally excluded from the multivariable prediction model because the final pathological diagnosis becomes available only after surgery and therefore cannot contribute to preoperative or intraoperative risk prediction. Moreover, postoperative histopathological findings are intrinsically related to both the extent of surgery performed and the preoperative FNAB results, creating substantial collinearity among these variables. Therefore, the multivariable model incorporated preoperative FNAB cytology together with surgical procedure, as these variables are available before or during surgery and therefore have greater clinical applicability for perioperative risk stratification.

Unexpectedly, the number of incidentally excised parathyroid glands identified on final histopathological examination was not significantly associated with symptomatic hypocalcemia. Although numerous previous studies have identified incidental parathyroidectomy as an important risk factor for postoperative hypocalcemia or transient hypoparathyroidism [15,16,21,26,28], our findings suggest that early symptomatic hypocalcemia may not be determined solely by the number of inadvertently excised glands. Consistent with our findings, Emmanouilidou et al. [23] reported in 2025 that incidental parathyroid excision was not independently associated with postoperative hypocalcemia. Preservation of parathyroid function depends not only on maintaining the glands in situ but also on preserving their vascular supply. Consequently, devascularization, ischemia, thermal injury, postoperative edema, and transient impairment of parathyroid perfusion may contribute more substantially to early symptomatic hypocalcemia than inadvertent gland excision alone. Moreover, histopathological identification of excised parathyroid tissue does not necessarily reflect the functional capacity of the remaining glands. The retrospective design of the present study should also be considered when interpreting these findings, because intraoperative assessment of parathyroid viability and vascular integrity was not available. Therefore, our observations regarding incidental parathyroidectomy should be regarded as exploratory rather than definitive. Accordingly, our results should not be interpreted as minimizing the importance of meticulous parathyroid preservation. Rather, they emphasize that symptomatic hypocalcemia is a multifactorial outcome in which preservation of parathyroid vascularization and functional integrity may be more important than the absolute number of glands remaining in situ or inadvertently excised.

Several clinical implications emerge from the present findings. Patients undergoing bilateral total thyroidectomy with central neck dissection and those with lower preoperative serum calcium concentrations should be considered at increased risk of symptomatic hypocalcemia and may benefit from closer postoperative surveillance and individualized calcium supplementation. Conversely, patients without these risk factors may be suitable candidates for earlier discharge. Distinguishing symptomatic from asymptomatic hypocalcemia may also prevent unnecessary treatment of isolated biochemical abnormalities.

Although albumin and PTH were intentionally left out of the multivariable model to avoid overadjustment because they are involved in the process of regulating calcium levels, the unique patterns seen in the post hoc pairwise comparisons need to be highlighted. In univariate analyses, postoperative albumin and PTH differed significantly among the three groups. Pairwise comparisons showed that patients with normocalcemia had higher postoperative albumin levels than both patients with asymptomatic and symptomatic hypocalcemia, suggesting that lower postoperative albumin may accompany postoperative biochemical hypocalcemia but does not appear to distinguish symptomatic from asymptomatic patients. In contrast, postoperative PTH showed a distinct pattern: it did not differ significantly between normocalcemic and asymptomatic patients but was significantly lower in the symptomatic hypocalcemia group. This finding suggests that early postoperative PTH may provide more specific information regarding clinically relevant parathyroid dysfunction and may help identify patients at increased risk of symptomatic hypocalcemia. Therefore, postoperative PTH may be useful for assessing early risks and monitoring patients after thyroidectomy. Although this finding should be considered exploratory given the retrospective design of the present study and the absence of serial postoperative PTH measurements, it is supported by the recent literature. In 2025, Lee et al. [29] reported that postoperative PTH decline was a predictive factor for symptomatic hypocalcemia. More recently, Youssef et al. [30] reported in 2026 that PTH measured during the first postoperative hour showed high sensitivity for detecting post-thyroidectomy hypocalcemia. These findings, together with our observation that postoperative PTH specifically distinguished symptomatic hypocalcemia from both normocalcemia and asymptomatic biochemical hypocalcemia, suggest that early postoperative PTH measurement may have clinical value in identifying patients at risk of symptomatic hypocalcemia.

Post-thyroidectomy complications may include, but are not limited to, hypocalcemia, hypoparathyroidism, recurrent laryngeal nerve injury, postoperative bleeding, and injury to the airway, esophagus, or thoracic duct. Consequently, meticulous anatomical preservation, vigilant postoperative monitoring, and timely management are imperative [31]. A recent comprehensive review emphasized the roles of parathyroid injury, vascular compromise, perioperative PTH, vitamin D, and magnesium status in postoperative calcium disturbances, highlighting the potential value of early PTH assessment for timely management [31]. Furthermore, the optimal extent of thyroidectomy, particularly in differentiated thyroid cancer, remains a subject of debate. Studies comparing total and subtotal thyroidectomy have reported inconsistent findings regarding postoperative morbidity, with some studies showing no significant difference in early complications, whereas systematic evidence suggests a higher risk of transient hypocalcemia following total thyroidectomy [32,33]. More recent evidence comparing total thyroidectomy with completion thyroidectomy has generally indicated a higher risk of transient hypocalcemia following total thyroidectomy, while differences in permanent hypocalcemia, recurrent laryngeal nerve injury, and postoperative hematoma appear less consistent [34]. However, the present study was not designed to directly address this controversy: differentiated thyroid cancer was not analysed as a separate subgroup, total thyroidectomy was not compared specifically with subtotal thyroidectomy, and postoperative complications were not assessed as a composite outcome. Rather, our analysis focused specifically on symptomatic postoperative hypocalcemia and identified bilateral total thyroidectomy with central neck dissection as an independent predictor of this clinically relevant outcome. Thus, our findings should be interpreted within this specific clinical context rather than as evidence regarding the overall complication profile of total versus subtotal thyroidectomy. The selection of energy device may also have an impact on hemostasis and postoperative bleeding. A 15-year, single-center study comparing LigaSure and Harmonic Scalpel in 1653 total thyroidectomies reported no significant difference in major postoperative bleeding requiring reoperation. However, Harmonic Scalpel was associated with more favorable hemostatic outcomes, particularly among patients with thyroid carcinoma [35]. However, the present study was unable to perform this comparison due to the routine use of LigaSure in all thyroidectomies. Consequently, the potential impact of the hemostatic device on postoperative complications could not be independently evaluated.

The present study has several strengths. First, unlike most previous investigations, patients were categorized into three clinically meaningful postoperative calcium groups rather than being dichotomized simply as hypocalcemic or normocalcemic. This approach enabled direct comparison of patients with asymptomatic biochemical hypocalcemia and symptomatic hypocalcemia, providing a more clinically relevant assessment of postoperative calcium disturbances. Furthermore, by focusing the multivariable analysis on symptomatic hypocalcemia rather than isolated biochemical abnormalities, our findings may have greater applicability to postoperative patient management, risk stratification, and discharge planning. Second, preoperative USG findings, FNAB cytology, definitive histopathological diagnoses, biochemical parameters, and surgical characteristics were evaluated simultaneously within a single cohort, enabling comprehensive assessment of established and less extensively investigated predictors of postoperative calcium disturbances. Third, the multivariable regression model was deliberately restricted to variables available before or during surgery, thereby identifying independent predictors that may realistically contribute to perioperative risk stratification and clinical decision-making.

Several limitations of the present study should be acknowledged. First, the retrospective design inevitably introduces the possibility of selection bias and dependence on the accuracy and completeness of existing medical records. Although all patients underwent systematic twice-daily postoperative clinical assessment during the first 72 h and postoperative symptoms were routinely documented using a standardized institutional protocol, the retrospective nature of the study may still have introduced information and documentation bias. Mild hypocalcemic symptoms might not have been reported by patients or documented by healthcare staff despite the standardized assessment protocol, potentially resulting in misclassification of some symptomatic patients as asymptomatic. In addition, the retrospective design precluded evaluation of symptom severity, response to calcium supplementation, and the duration of postoperative hypocalcemia. Consequently, refractory or persistent symptomatic hypocalcemia could not be analyzed as a separate clinical entity. Although patients with electrolyte disturbances requiring preoperative treatment were excluded and no routine calcium, magnesium, or vitamin D supplementation was administered perioperatively, the retrospective design did not allow reliable assessment of over-the-counter multivitamin, mineral, calcium, or vitamin D supplement use. Other medications potentially affecting calcium homeostasis were also not systematically captured. Unrecorded supplement use or medication exposure may therefore have contributed to residual confounding in the assessment of postoperative calcium homeostasis. Future prospective studies incorporating standardized symptom scores, treatment response, and systematic assessment of medication and supplement use are warranted to further refine postoperative risk stratification.

Second, this was a single-center study with a relatively limited sample size, which may have reduced statistical power for some subgroup analyses and may limit the generalizability of the findings to other institutions with different patient populations and surgical practices. Third, postoperative calcium status was evaluated only during the early postoperative period; therefore, permanent hypoparathyroidism and long-term calcium homeostasis were beyond the scope of the present investigation. Fourth, perioperative vitamin D and magnesium concentrations were unavailable and therefore could not be incorporated into the analyses. Given the complex physiological interactions among vitamin D, calcium, magnesium, and PTH, the absence of these variables precluded a more comprehensive evaluation of calcium homeostasis after thyroidectomy. Fifth, the specific clinical indications for central neck dissection were not systematically available as an individual variable in the retrospective records and therefore could not be directly incorporated into the analysis. Although preoperative ultrasonographic findings, FNAB cytology, and surgical indications were available and evaluated in relation to postoperative calcium status, the specific rationale for performing central neck dissection could not be separately assessed. Sixth, patients who underwent intraoperative parathyroid autotransplantation were excluded because this procedure may independently influence postoperative calcium homeostasis. This exclusion may have introduced selection bias and could have led to underestimation of the overall risk of postoperative hypocalcemia, particularly in patients undergoing more complex thyroid surgery. Seventh, although incidentally excised parathyroid glands were identified histopathologically, objective assessment of parathyroid vascularization or viability was not routinely available. Because preservation of parathyroid function depends on vascular integrity as much as gland preservation itself, this limitation may partially explain the lack of association between incidental parathyroidectomy and symptomatic postoperative hypocalcemia observed in the present study. Finally, a multivariable analysis restricted to patients with biochemical hypocalcemia, comparing symptomatic with asymptomatic patients, was not performed, and therefore factors specifically associated with the development of symptoms among hypocalcemic patients could not be assessed. Such an analysis would address a clinical question different from the primary objective of identifying predictors of symptomatic hypocalcemia in the overall postoperative cohort.

## 5. Conclusions

In conclusion, symptomatic postoperative hypocalcemia should be considered a distinct clinical outcome rather than solely a manifestation of biochemical hypocalcemia. Bilateral total thyroidectomy with central neck dissection and lower preoperative serum calcium concentrations independently predicted symptomatic postoperative hypocalcemia, whereas preoperative USG characteristics, FNAB cytology, final histopathological diagnosis, and incidental parathyroidectomy were not independent predictors. These findings suggest that readily available preoperative and intraoperative variables may help identify patients at increased risk of symptomatic hypocalcemia following thyroidectomy and support risk-stratified postoperative monitoring.

## Figures and Tables

**Table 1:** Baseline Surgical Characteristics According to Postoperative Calcium Status.

Surgical Characteristics (*n* = 245)		Normocalcemia (*n* = 88)	Asymptomatic Hypocalcemia (*n* = 87)	Symptomatic Hypocalcemia (*n* = 70)	*p*
Sex	Female	68 (36.4%)	67 (35.8%)	52 (27.8%)	0.892
	Male	20 (34.5%)	20 (34.5%)	18 (31%)	
Operation Type	Bilateral Total Thyroidectomy	54 (36.7%)	57 (38.8%)	36 (24.5%)	0.001
	Unilateral Total Thyroidectomy (Hemithyroidectomy)	18 (54.5%)	12 (36.4%)	3 (9.1%)	
	Completion Thyroidectomy	5 (35.7%)	3 (21.4%)	6 (42.9%)	
	Bilateral Total Thyroidectomy with Central Neck Dissection	11 (21.6%)	15 (29.4%)	25 (49%)	
Indication	Multinodular Goiter	46 (44.2%)	36 (34.6%)	22 (21.2%)	0.061
	Hyperthyroidism	4 (30.8%)	5 (38.5%)	4 (30.8%)	
	Obstructive/Substernal Goiter	0	3 (100%)	0	
	Papillary Thyroid Carcinoma	20 (27%)	23 (31.3%)	31 (41.9%)	
	AUS/FLUS	6 (42.9%)	5 (35.7%)	3 (21.4%)	
	Follicular Thyroid Carcinoma or Suspicion	6 (28.6%)	11 (52.4%)	4 (19%)	
	Other	6 (37.5%)	4 (25%)	6 (37.5%)	
Drain Placement	No	3 (33.3%)	4 (44.4%)	2 (22.2%)	0.836
	Yes	85 (36.0%)	83 (35.2%)	68 (28.8%)	

*p*: statistical significance value, *n*: number of patients, AUS/FLUS: Atypia of Undetermined Significance/Follicular Lesion of Undetermined Significance. Data are presented as *n* (%). *p* values were calculated using the Chi-square test or Fisher’s exact test, as appropriate.

**Table 2:** Comparison of Preoperative and Early Postoperative Biochemical Parameters According to Postoperative Calcium Status.

Biochemical Parameters (*n* = 245)	Normocalcemia (*n* = 88)	Asymptomatic Hypocalcemia (*n* = 87)	Symptomatic Hypocalcemia (*n* = 70)	*p*
Preoperative TSH	2.77 (2.51) (0.62–13.3)	0.91 (2.78) (0–6.9)	0.97 (1.12) (0.27–5.15)	0.238
Preoperative T3	3.48 (0.6) (2.57–4.06)	3.18 (0.53) (2.89–4.22)	3.36 (0.57) (2.83–4.59)	0.303
Preoperative T4	0.76 (0.14) (0.44–0.95)	0.71 (0.31) (0.44–1.09)	0.94 (0.3) (0.66–1.12)	0.289
Preoperative Anti-TPO	3.45 (38.95) (0.2–569.3)	1.55 (61.4) (0.3–884)	0.6 (6.2) (0.2–708.9)	0.978
Preoperative Albumin	4.3 (0.4) (3.9–4.93)	4.2 (0.55) (3.5–4.7)	4.1 (0.65) (3.3–4.8)	0.219
Preoperative Calcium	9.65 (0.48) (8.9–10.2)	9.43 (0.83) (8.7–10.1)	9.4 (0.75) (8.4–10.2)	0.018
Preoperative Creatinine	0.66 (0.12) (0.47–0.91)	0.65 (0.17) (0.36–1.12)	0.57 (0.17) (0.45–0.79)	0.633
Preoperative PTH	55.8 (19.85) (30.1–230.4)	47.3 (31.15) (27–147)	48.3 (33.6) (22–142)	0.310
Postoperative Albumin	3.95 (0.34) (1.02–5.08)	3.8 (0.42) (3.2–4.2)	3.7 (0.35) (2.2–4.1)	0.002
Postoperative PTH	43.6 (30) (2–110.4)	33.25 (22.9) (11.8–133.1)	11.9 (37.65) (0.8–56.1)	<0.001

*p*: statistical significance value, *n*: number of patients, TSH: thyroid-stimulating hormone, T3: free triiodothyronine, T4: free thyroxine, Anti-TPO: anti-thyroid peroxidase antibody, PTH: parathyroid hormone. Data are presented median (interquartile range [IQR]; minimum–maximum). *p* values were calculated using the Kruskal–Wallis test.

**Table 3:** Post Hoc Pairwise Comparisons of Biochemical Parameters Among the Study Groups.

Biochemical Parameter	Normocalcemia vs. Asymptomatic Hypocalcemia	Normocalcemia vs. Symptomatic Hypocalcemia	Asymptomatic Hypocalcemia vs. Symptomatic Hypocalcemia
Preoperative Calcium	0.021	0.011	0.662
Postoperative Albumin	0.013	0.001	0.253
Postoperative PTH	0.178	<0.001	<0.001

PTH, parathyroid hormone. Values represent *p* values from post hoc pairwise comparisons using the Mann–Whitney U test with Bonferroni correction following a significant Kruskal–Wallis test.

**Table 4:** Comparison of Preoperative Ultrasonographic Characteristics According to Postoperative Calcium Status.

Preoperative Ultrasonography Findings (*n* = 227)		Normocalcemia (*n* = 83)	Asymptomatic Hypocalcemia (*n* = 78)	Symptomatic Hypocalcemia (*n* = 66)	*p*
Nodule Size	<1 cm	10 (32.3%)	15 (48.4%)	6 (19.4%)	0.246
	1–2 cm	32 (44.4%)	18 (25%)	22 (30.6%)	
	2–4 cm	31 (35.6%)	30 (34.5%)	26 (29.9%)	
	>4 cm	10 (31.3%)	13 (40.6%)	9 (28.1%)	
	Extrathyroidal invasion	0	2 (40%)	3 (60%)	
Nodule Character	Cystic	5 (35.7%)	4 (28.6%)	5 (35.7%)	0.727
	Mixed	32 (41%)	23 (29.5%)	23 (29.5%)	
	Solid	46 (34.1%)	51 (37.8%)	38 (28.1%)	
Calcification	None	47 (39.5%)	40 (33.6%)	32 (26.9%)	0.846
	Macro	23 (33.8%)	24 (35.3%)	21 (30.9%)	
	Peripheral Rim	4 (22.2%)	7 (38.9%)	7 (38.9%)	
	Punctate Echogenic	9 (40.9%)	7 (31.8%)	6 (27.3%)	
Parenchymal Echogenicity	Anechoic	5 (26.3%)	12 (63.2%)	2 (10.5%)	0.102
	Hyperechoic/Isoechoic	23 (39.7%)	21 (36.2%)	14 (24.1%)	
	Hypoechoic	54 (37.2%)	43 (29.7%)	48 (33.1%)	
	Markedly Hypoechoic	1 (20%)	2 (40%)	2 (40%)	
Margin	Smooth	78 (36.8%)	74 (34.9%)	60 (28.3%)	0.627
	Lobulated/irregular	5 (33.3%)	4 (26.7%)	6 (40%)	
TI-RADS Score		4 (2) (0–9)	4 (2) (0–8)	4 (2) (2–9)	0.341

*n*: number of patients, *p*: statistical significance value. Categorical variables are expressed as frequency (percentage) and were compared using Pearson’s chi-square test or Fisher’s exact test, as appropriate. Continuous variables are presented as median (IQR; minimum–maximum) and were compared using the Kruskal–Wallis test.

**Table 5:** Comparison of Preoperative Fine-Needle Aspiration Biopsy Cytology According to Postoperative Calcium Status.

Preoperative FNAB Findings (*n* = 198)		Normocalcemia (*n* = 88)	Asymptomatic Hypocalcemia (*n* = 87)	Symptomatic Hypocalcemia (*n* = 70)	*p*
FNAB Results, Based on Bethesda Classification	Nondiagnostic	5 (62.5%)	2 (25%)	1 (12.5%)	0.047
	benign	34 (41%)	31 (37.3%)	18 (21.7%)	
	AUS/FLUS	9 (42.9%)	4 (19%)	8 (38.1%)	
	Follicular Thyroid Carcinoma or Suspicion of Follicular Neoplasm	7 (35%)	10 (50%)	3 (15%)	
	Suspicion of Malignancy	10 (50%)	6 (30%)	4 (20%)	
	Malignant	10 (21.7%)	15 (32.6%)	21 (45.7%)	
FNAB Result	Low-Risk (I–II)	39 (42.9%)	33 (36.3%)	19 (20.9%)	0.126
	High-Risk (III–VI)	36 (33.6%)	35 (32.7%)	36 (33.6%)	

*p*: statistical significance value, *n*: number of patients, AUS/FLUS: Atypia of Undetermined Significance/Follicular Lesion of Undetermined Significance. Data are presented as *n* (%). *p* values were calculated using the Chi-square test or Fisher’s exact test, as appropriate.

**Table 6:** Comparison of Final Histopathological Diagnoses and Incidentally Excised Parathyroid Glands According to Postoperative Calcium Status.

		Normocalcemia (*n* = 88)	Asymptomatic Hypocalcemia (*n* = 87)	Symptomatic Hypocalcemia (*n* = 70)	*p*
Final Histopathological Diagnosis	Benign Nodular Disease	46 (41.8%)	39 (35.5%)	25 (22.7%)	0.020
	Thyroiditis	3 (50%)	2 (33.3%)	1 (16.7%)	
	Benign Neoplasm	6 (50%)	5 (41.7%)	1 (8.3%)	
	Papillary Thyroid Carcinoma	29 (27.4%)	38 (41.7%)	39 (36.8%)	
	Medullary Thyroid Carcinoma	0	1 (20%)	4 (80%)	
	Other Malignant Tumors	4 (66.7%)	2 (33.3%)	0	
Final Histopathological Diagnosis	Benign	55 (43%)	46 (35.9%)	27 (21.2%)	0.012
	Malignant	33 (28.2%)	41 (35%)	43 (36.8%)	
Number of Parathyroid Glands on Pathologic Examination	0	48 (41.4%)	39 (33.6%)	29 (25%)	0.131
	1	39 (32.8%)	45 (37.8%)	35 (29.4%)	
	≥2	1 (10%)	3 (30%)	6 (60%)	

*p*: statistical significance value, *n*: number of patients. Data are presented as *n* (%). *p* values were calculated using the Chi-square test or Fisher’s exact test, as appropriate.

**Table 7:** Multivariable Logistic Regression Analysis for Symptomatic Postoperative Hypocalcemia.

Variable	Adjusted OR	95% CI	*p* Value
Unilateral Thyroidectomy	Reference	–	–
Bilateral Total Thyroidectomy	3.21	0.89–11.66	0.076
Completion Thyroidectomy	4.40	0.53–36.48	0.170
Bilateral Total Thyroidectomy with Central Neck Dissection	6.63	1.68–26.11	0.007
Preoperative Serum Calcium (Per 1 mg/dL Increase)	0.46	0.22–0.94	0.036
FNAB Result (High-Risk vs. Low-Risk Category)	1.42	0.67–3.01	0.363

OR: odds ratio, CI: confidence interval, *p*: statistical significance value, FNAB: fine-needle aspiration biopsy. Unilateral thyroidectomy was used as the reference category for surgical procedure, and the low-risk FNAB category (Bethesda I–II) served as the reference category for cytological risk classification. Adjusted ORs were obtained using the enter method in binary logistic regression.

## Data Availability

The datasets generated and analyzed during the current study are stored in a secure institutional repository and are not publicly available because they contain sensitive personal health information protected under the Turkish Personal Data Protection Law (Law No. 6698; KVKK), the regulations of the Republic of Türkiye Ministry of Health, and the conditions of the institutional ethics approval. De-identified data may be made available by the corresponding author upon reasonable request and subject to approval by the institutional ethics committee and applicable legal requirements.

## Abbreviations

The following abbreviations are used in this manuscript:

FNAB	fine-needle aspiration biopsy
STROBE	Strengthening the Reporting of Observational Studies in Epidemiology
PTH	parathyroid hormone
TSH	thyroid-stimulating hormone
T3	free triiodothyronine
T4	free thyroxine
Anti-TPO	anti-thyroid peroxidase antibody
TI-RADS	Thyroid Imaging Reporting and Data System
AUS/FLUS	atypia of undetermined significance/follicular lesion of undetermined significance
IQR	interquartile range
OR	Odds ratio
CI	Confidence interval
USG	ultrasonography
